# Toward a Theory of Coexistence in Shared Social-Ecological Systems: The Case of Cook Inlet Salmon Fisheries

**DOI:** 10.1007/s10745-016-9806-0

**Published:** 2016-01-23

**Authors:** Philip A. Loring

**Affiliations:** School of Environment and Sustainability, University of Saskatchewan, Saskatoon, SK Canada

**Keywords:** Coexistence, Common pool resources, Conflict, Fisheries, Resilience, Sustainability, Alaska

## Abstract

Coexistence theory (CT) in community ecology provides a functional perspective on how multiple competing species coexist. Here, I explore CT’s usefulness for understanding conflict and coexistence among human groups with diverse livelihood interests in shared resources such as fisheries. I add three concepts from social science research on coexistence: adaptability, pluralism, and equity and apply this expanded theoretical framework to the case of salmon fisheries in Alaska’s Cook Inlet, synthesizing catch records with anthropological research. The analysis addresses issues of inequity, such as who bears the costs of conservation measures, a lack of pluralism, in that people have come to devalue their neighbors, and a decline in resilience for some sectors, all of which undermine the likelihood of these groups continuing coexistence. I discuss policy options for addressing escalating conflict in the region, such as improving equity in management and the resilience of some fishing groups to temporary closures. Finally, I discuss points of engagement for CT with other areas of sustainability science such as resilience thinking.

## Introduction

In any community there are invariably multiple groups that hold different values and priorities for ecosystems and the many services that they provide. Often, these conflict: different user groups in a shared fishery, for example, often dispute the fairness and wisdom of allocations, quotas, and other actions taken to ensure the sustainability of the resource (Hilborn 2007; Pomeroy *et al.*^2007^; Loring and Gerlach 2010). Yet biocultural diversity is important both to the people whose identity and self-worth is intertwined with the place-based nature of their livelihoods (such as small-scale fishers), and also in contributing to local and regional resilience and sustainability (Maffi 2001; Turner *et al.*^2003^; Cocks 2006; Nabhan 2012; Leslie and McCabe 2013). Thus, understanding how bioculturally diverse groups of people can coexist despite the potential for conflict is an important area for research.

Conflicts over shared resources can seem inevitable to the people involved, but in many cases conflicts are essentially fostered by aspects of resource governance (Nie 2003); market-based regimes such as tradable fishing quotas, for example, can favor industrialized modes of resource extraction at the expense of traditional and artisanal systems: smallholders are often driven out by larger competitors (McCay 1995; Carothers 2010), or forced to compete with one another over what remains (Loring 2013; Jenkins 2015). Conversely, there are numerous examples where people with diverse but overlapping livelihood strategies coexist sustainably on shared and resource-limited landscapes (Barth 1956; Braroe 1965; Bennett 1969; Harris 1974; Masuda *et al.*^1985^; Kassam 2010). Understanding these cases, and developing a theory of what makes them possible, would be significant steps towards managing natural resource conflicts to ensure both environmental sustainability and social justice (Loomis 2000; Maffi 2001; Redpath *et al.*^2013^).

I explore these issues of conflict and coexistence in the case of salmon fisheries in Alaska’s Upper Cook Inlet (UCI). I draw on coexistence theory (CT) (Chesson 2000a, 2000b) as a starting point for developing a theory of coexistence of diverse groups in a shared resource setting. I add three concepts drawn from social science research on coexistence, specifically: adaptability, pluralism, and equity, and discuss some of the challenges inherent to adapting natural science concepts for use in anthropological contexts. I use this expanded theoretical framework, which I term social-ecological coexistence theory (SCT), to tease apart the ongoing “salmon wars” in Alaska. SCT draws attention to many of the reasons why the diverse fishing groups have coexisted in the UCI for decades, but also to differences of resilience and inequity that currently threaten this coexistence. Likewise, SCT aids in the identification of possible solutions to the worsening conflict in the UCI—specifically, ways to increase equity and resilience and flexibility of stakeholder groups. I conclude by discussing some of SCT’s potential complementarities with other areas of sustainability science such as resilience thinking and commons research.

## Background

Why so much diversity exists in the world and how competing species come to coexist in space and time are founding questions of community ecology (Elton 1951; MacArthur 1955; Hutchinson 1959). Biologically diverse systems are generally more productive than less diverse ones (Tilman *et al.*^1996^), but diversity does not guarantee stability (De Angelis 1975; Lawler and Morin 1993; Naeem 2002). Over time, dominant species displace and exclude weaker species, and species’ niches become differentiated through adaptation and niche construction processes (Odling-Smee *et al.*^1996^). Yet, there are also examples where groups of species with relatively similar niches (i.e., guilds) coexist stably.

CT uses the principle of limited similarity, which states that multiple species cannot coexist without an ability to exploit sufficiently unique niches, to explain these examples of inter-species coexistence (Macarthur and Levins 1967; Abrams 1983; Chesson 2000b). In cases of direct competition, coexistence is often achieved through spatial or temporal partitioning of resources or habitat (Chesson 2000a; Velázquez *et al.*^2014^). Limited similarity is necessary but not sufficient to achieving stable coexistence, however, because a species’ realized niche, the niche it currently occupies, is not necessarily coterminous with its actual niche, the range of conditions within which it can survive (Hutchinson 1965). While two competing species may coexist effectively when both populations are healthy, one species may displace the second if it is weakened by a chance event. CT therefore also posits two kind of coexistence, stable and unstable, i.e., whether there are factors that limit competition and foster resilience in the case of a chance event such that the impacted species is able to recover despite the presence of competitors.

CT also identifies two functional mechanisms by which coexistence is achieved: stabilizing and equalizing mechanisms. Stabilizing mechanisms are factors that prevent one species from gaining an advantage over the second, for example, density-dependent predation, where a predator switches from targeting one prey species (A) to another (B) when the population of the first declines (and vice versa). Equalizing mechanisms reduce fitness differences among species. For example, if prey species A is a better competitor than B, but a predator has a stronger preference for A over B, the predator will effectively equalize B’s disadvantage. Similarly if species B has alternative food or habitat options, this also equalizes the competitive differential in addition to providing resilience to B in the case of a disturbance to its primary food source, which as noted is also requisite to stable coexistence.

In sum, CT sets out the mechanisms by which similarities in niche and competition among species are limited, such that: 1) none of the coexisting species will be displaced over time, and 2) each remains resilient to chance events. If species differ greatly in average fitness, then strong mechanisms are necessary for them to coexist stably, and if fitness differences are small, weaker and more indirect mechanisms are generally sufficient to allow long-term coexistence (Chesson 2000a).

## Coexistence in Social-Ecological Systems

Coexistence of cultural groups with diverse yet overlapping niches is well documented in the social sciences (Barth 1956; Bennett 1969; Masuda *et al.*^1985^; Kassam 2010). This literature offers some additional concepts to the present discussion, including pluralism, adaptability, and equity, each of which enriches CT’s usefulness in linked, social-ecological settings (Table 1).

**Table 1:** Proposed principles of social-ecological coexistence theory

Principle	Description	Noteworthy Citations
Limited Similarity	Niche differentiation, for example commercial fishers targeting different species than sport fisheries	(Barth 1956; Hardin 1960; Macarthur and Levins 1967; Abrams 1983)
Limited Competition	Stabilizing and equalizing mechanisms (below) limit competition such that groups are not displaced and are able to recover if impacted by some external factor.	(Paine 1966; Quinn 1991; Peteraf 1993)
Stabilizing Mechanism	Prevents one group from gaining or exploiting a competitive advantage over others, for example policies that limit growth of any specific sector	(Paine 1966; Holling 1973; Chesson 2000a)
Equalizing Mechanism	Reduces competitive advantage, for example policies that limit downstream fishing to ensure passage to upstream users	(Chesson 2000a)
Resilience	An equalizing mechanism that is essential for stable coexistence because it keeps a group from being displaced if temporarily impacted by a chance event.	(Holling 1973; Pimm 1984; Folke *et al.* 2010)
Adaptability	Whether people have the flexibility to experiment and innovate.	(Bennett 1969; Moran 1979; Bates 2004; Folke *et al.* 2010)
Pluralism	People value biocultural diversity, and this serves as a stabilizing and/or equalizing mechanism.	(Kassam 2010; Karner and Parker 2011)
Equity	An equalizing mechanism, where social mores and institutions ensure equitable outcomes and preclude competitive displacement.	(Sen 1983; Lam and Pitcher 2012)

### Barth on Coexistence

Perhaps the most well-known study of coexistence in social-ecological systems is Barth’s (1956) study of ecological relationships among three ethnic groups in the Swat region of Pakistan. His explanation for how the Pathans, sedentary agriculturalists who live in the lower-altitude valley, the Kohistanis, who practice both agriculture and transhumant herding in higher-altitude regions, and the Gujars, herders who live in both The Valley and mountain regions, have come to coexist in a shared landscape rests largely on the concepts of niche differentiation and competitive displacement: the Pathans consider higher altitude environs to be uninhabitable; the Kohistanis, however, make a living in the high latitude regions by complementing agriculture with herding. Because the Pathans are stronger militarily, the Kohistanis refrain from attempting to colonize The Valley. The third group, the Gujars, are able to subsist in both the Pathan-occupied region and the western-half of the Kohistani region through social flexibility: in the former, they practice transhumant herding as serfs to the Pathans in a semi-mountainous region that the Pathans control but consider useless. In the mountains, the Gujars are fully nomadic herders in areas amendable to herding but unsuited to Kohistani agriculture. Individual Gujars are also flexible, and willing to move from one region and strategy to the other.

Barth concludes that diverse groups can coexist provided they can sufficiently differentiate themselves from each other and especially if they can forge some sort of mutualistic economic or ecological relationship; otherwise, he argues, conflict and displacement are inevitable, with the ‘weaker’ parties eventually being forced to either less desirable environs or eliminated altogether (cf. Love 1977; Orlove 1980).

### Adaptability

Bennett’s stated goal in *Northern Plainsmen* (1969) is to explore relations among the Plains Cree, Hutterites, wheat farmers, and cattle ranchers in an area of Southwest Saskatchewan that allow for “alternative ways of functioning within the same general framework of natural and economic resources” (p. 18). Rather than focusing on niche in a deterministic sense, Bennett uses the language of adaptation and what he calls the ‘adaptive process’: how people adjust and experiment with their adaptive strategies, and how these adjustments come to be codified in practice over time. He describes a co-evolutionary milieu among the farming and ranching groups and notes that while relatively stable, the “regional culture has not yet found a balance between cooperation and competition” (p. 324).

Both Barth and Bennett highlight how coexistence is a process, with innovation and adaptation often essential to long-term stability (Bates 2004; Cumming and Collier 2005; Loring 2007; Folke *et al.*^2010^). As Bennett argues, not even the most ‘traditional’ of societies are ever truly stable (Bennett 1976). Thus, behavioral or cultural change, or even movement of some people from one group to another, should not be mistaken for evidence that groups are not persisting and coexisting. The critical determining factor is whether these changes occur from within a context of self-determination and agency, or whether they are forced by sociopolitical or ecological circumstances.

### Pluralism

People’s ability to rapidly adopt new strategies might also serve to destabilize regional coexistence if a new strategy changes the competitive balance. Social-ecological coexistence hinges also on whether people value pluralism in their communities (Kassam 2010). Indeed, Bennett noted the importance of pluralism to coexistence in the social function of rodeos as a shared cultural experience. More recently, Kassam (2010) in his study of the various competing culture groups in the Pamir Mountains of Afghanistan defines pluralism as the product of social institutions through which people come to value and even prioritize cultural diversity:

The case of the Kyrgyz and Wakhi or the Pashtu and Shugni is informative because their milieu is rife with conflict, yet their approach is pragmatic as they negotiate human-ecological relations that help secure their livelihoods through the practice of pluralism. The interdependence between the Kyrgyz and Wakhi or the Pashtu and Shugni is ….. not only an outcome of a materially determined calculus, but an organic engagement of diverse cultural systems and social structures in the context of varied but overlapping ecological zones. Trust and confidence sustain this interdependence (p.14).

Pluralism, where held and practiced as a cultural value, thus represents a special kind of equalizing mechanism through which competition among groups is limited because each group accepts and values the others’ right to exist within the same ecological space. Pluralism is especially relevant for social-ecological systems because unlike most other species (though there are some exceptions), humans are known to systematically eliminate some or even all of their competitors, i.e., wage war (Vayda 1976; Quinn 1991). The near-extirpation of coyotes and wolves in the US is one example; the forced displacement of Native Americans is another. Such actions are ideological, and pluralism as a cultural value is antithetical to this ideology of competition through extirpation.

### Equity

It is generally recognized that equity is an essential prerequisite to the coexistence of diverse social groups; inequity is widely understood to be a cause of conflict and unsustainable behavior (Sen 1983; Nayak *et al.*^2014^); when social institutions attend to equity in how resources are managed, those resources are more sustainable: users are more compliant with rules, have greater trust in management regimes, and have less reason to view competing sectors as a threat (Bennett *et al.*^2001^; Bundy *et al.*^2008^; Lam and Pitcher 2012). For ecosystem-based management to be considered equitable, they must go further than simply managing a resource for some targeted yield and work toward such goals as recognizing the intrinsic value of non-human aspects of ecosystems and restoring and preserving ecosystem integrity and resilience (Chapin *et al.*^2011^; Lam and Pitcher 2012).

In sum, I propose a theoretical framework for social-ecological coexistence summarized as follows: groups of people with competing interests in a shared ecosystem can coexist sustainably if direct competition is limited, and if competitive advantage is equalized (Table 1). As in non-human ecological communities, this involves both practical aspects of each group’s niche such as how, when, and where they harvest or otherwise enjoy shared resources; however, sociocultural institutions and values can also serve to limit competition and competitive advantage even in cases where the groups’ niches overlap, specifically whether the people involved value cultural pluralism, whether formal and informal social institutions ensure equity, and if people have the flexibility to adapt in the face of change.

### Words of Caution

It is important to note some of the challenges inherent to adapting concepts developed in the natural sciences for use in social science (Rhoades 1978). CT in community ecology is a highly mathematical and reductionist enterprise, which can be a concern given that social institutions and human agency defy reduction or transferability because they are deeply embedded in place, history, and culture (Friedman 1974; Cleaver 2002). For example, some set of local traditions or policies may provide resilience in one part of the world but drive conflict in another, depending on local cultural and political histories (Davidson 2010). Deterministic overtones are also of concern because natural science frameworks are not necessarily constructed with agency in mind. CT also generally focuses on interspecies dynamics, which may not always be directly analogous to dynamics among human groups or communities of practice.

Thus, the appropriateness of the ecological analogy must be evident and justified (Binford 2001). For this research, I argue that interspecies competition is sufficiently analogous to competition among fishing groups to justify the framework’s analytical power. Firstly, fishers in general and these fishers specifically are greatly attached to and invested in fishing as a lifestyle (Pollnac *et al.*^2001^; Pollnac *et al.*^2006^; Harrison 2013; Britton and Coulthard 2013). Likewise, in many places it is not easy for fishers to change how or where they fish, whether because of policy barriers to entering new fisheries or economic circumstances that keep them locked-in to their current ones (Cinner *et al.*^2009^; Carothers *et al.*^2010^).

## The Case Study

**Fig. 1:**
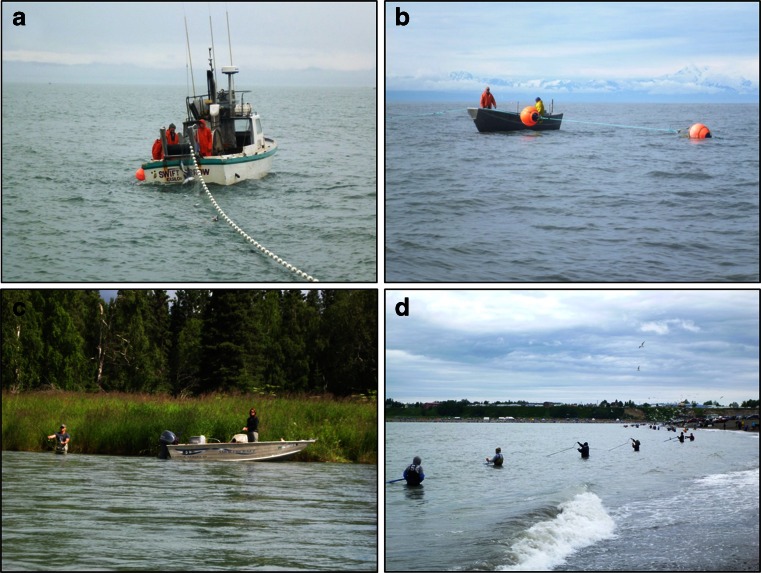
Salmon Fisheries of the Upper Cook Inlet and Kenai Peninsula. Examples of the competing fisheries in the region: **a** commercial drift fishing, **b** commercial set-net fishing, **c** sport-angling, **d** personal use dip-netting. Images originally published in (Loring *et al.* 2013). All photos by Philip Loring

Upper Cook Inlet (UCI) and the Kenai Peninsula, which bounds the Inlet on its eastern side, are known for their productive salmon fisheries, which involve all five species of Pacific salmon and support multiple commercial fishing fleets, an international sport fishery (including a large charter fishing sector), and personal use and subsistence fisheries open only to Alaskans. Commercial fishing (Fig. 1a, b) is the largest of the sectors in the region, and accounts for roughly 5 % of the total annual catch of salmon in Alaska (Shields and Dupuis 2013). Two distinct commercial fleets are active in the UCI: a “drift” fleet and a “set-net” fleet, both fish with gill nets and are managed as limited entry with tradable permits. Commercial fishing operations in the UCI are primarily a family affair run by in-state residents, many of whom live on the Kenai Peninsula, and it is not uncommon to find members from multiple generations of the same family fishing together (Harrison and Loring 2014).

Sport fishing (Fig. 1c) in the Kenai Peninsula is open access and popular. Many Alaskans rely on sport salmon fisheries for food, and guided sport fishing charters are big business, with the Kenai Peninsula and the Kenai River in particular widely regarded as a world-class sport fishing destination. According to industry data, visitors to the Kenai River account for over 1/3 of the total tourist anglers that visit Alaska (KRSA 2008). Many charter operators are state residents, but there is also a large contingent of summer workers from outside the state that work for local charter companies. The third sector, personal use “dip-net” fishing (Fig. 1d), is a food fishery that is open only to Alaska residents. Dip-net fisheries were introduced by the state in 1996 to replace a more disparate collection of personal use and subsistence practices and are immensely popular: for a short few weeks in July, Alaskans flock to the beaches of the Kenai and Kasilof Rivers to fish their limit, which is 25 fish for a single adult. For most Alaskans and especially those living in the Kenai Peninsula, dip-netting and sport fishing are the principle methods for procuring salmon for consumption, as local seafood is not sold in local grocery stores (Loring *et al.*^2012^). Many locals also obtain seafood through barter or as gifts directly from commercial fishers (ibid).

Each of the three fishing groups also has their own respective non-profit trade associations: commercial drift fishers are generally members of the United Cook Inlet Drift Association, set-netters are members of the Kenai Peninsula Fishermen’s Association, the Kenai River Sportfishing Association and the Kenai River Professional Guides association advocate for sport fishing, and at the time of my previous research a dip-net association was being formed. These groups are actively engaged in political debates over local conflicts, and are illustrative of how dedicated these fishers are to their respective fishing practices and ways of life.

In 2013 the commercial catch was roughly 4 million fish, whereas sport and personal use fisheries on the Kenai River both caught between 400,000 and 500,000 (Shields and Dupuis 2013). There are also a handful of smaller, so-called ‘educational’ subsistence fisheries throughout the region, though these are not party to the conflicts discussed below.

UCI salmon fisheries are notorious for long-standing and often rancorous conflict, specifically among commercial fishermen and sport fishermen (particularly those who run charter operations). At the crux of this conflict is a debate over how salmon catches should be allocated. Sport fishing advocates argue that in-river angling and the tourism that it supports brings more money to the region per fish caught than does commercial fishing, so that king salmon should be allocated for sport while commercial fleets should focus on sockeye salmon (Mayor’s Blue Ribbon Sportsmen Committee’s 2011). Though not the primary fished species for either sector, king salmon is the iconic and most sought after ‘trophy’ fish for sport anglers, and some sport boosters describe the catch of king salmon by commercial fishers as “bycatch” even though commercial fishing permits allow them to take all five species. Commercial fishers, conversely, hold that they have as much right to catch king salmon as sport fishers, regardless of which sector makes more money with the fish. They also contest the notion that the charter industry brings more economic benefits to the region than they do.

This conflict has remained non-violent over the last few decades, playing out primarily in policy debates and in the public sphere, but it has become troublingly acrimonious in the last few years (Harrison and Loring 2014). People openly dehumanize each other, there have been allegations of illegal spying, and most recently, sport sector advocates are actively pursuing legislation to eliminate the set-net fishery altogether. Some level of conflict among the sectors is perhaps not surprising, given the serial nature of these fisheries, and many locals reported not remembering a time when they did not fight over fish (Harrison and Loring 2014). Likewise, the various fisheries have ostensibly coexisted in the region for decades, even with sport and personal use fisheries growing significantly over the last 20 years (Fig. 3a, b). However, recent years of low king salmon abundance and a closure of both sport and set-net fishing in 2012 seem to have tipped this balance, driving a noteworthy escalation to the conflict and bringing into question whether or not these groups can continue to coexist.

I first provide additional data on the salmon fisheries drawn from previous ethnographic research in the region (Harrison 2013; Loring and Harrison 2013; Loring *et al.*^2013^, ^2014^; Harrison and Loring 2014). I present an analysis of catch data (Shields and Dupuis 2013, 2014), focusing on limited similarity among the fisheries, the presence of any stabilizing or equalizing mechanisms, whether fishing sectors are resilient and adaptable, and whether the fisheries are managed in a cultural context where pluralism is valued and equity protected.

### Limited Similarity

**Fig. 2:**
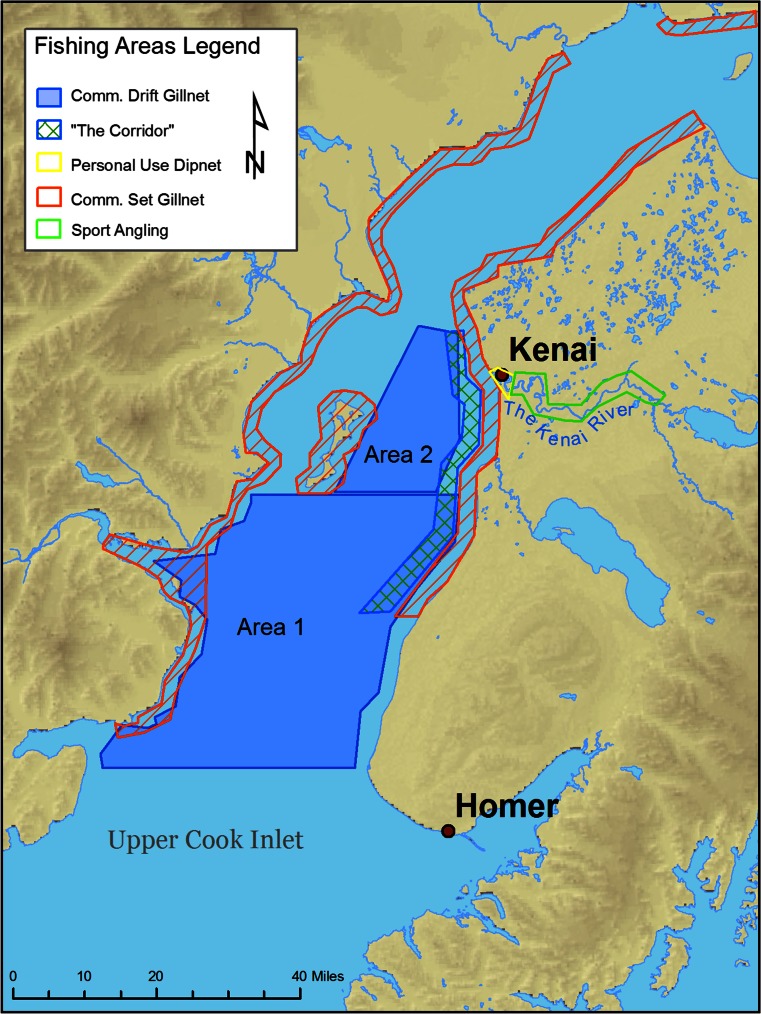
Geographic ranges of Upper Cook Inlet salmon fisheries. The fisheries of the UCI are serial in nature, which can foster conflict among users but also provides some limited similarity among the groups. Drift fishers have the widest range and flexibility, though fishing is sometimes restricted to the “corridor” to allow passage of fish to rivers further north

The fisheries vary notably in terms of the gear and fishing methods employed (Fig. 1a-d) and the locations where harvest occurs (Fig. 2). However, because these fisheries occur in serial, there is the possibility that a downstream group will create a bottleneck that reduces the success of upstream fishers, and this is ostensibly at the crux of the conflict. The existing management system currently works to avoid this problem; generally, fishing openings and closures are used to ensure escapement of sufficient salmon to spawning grounds, but strategic closures are also used to ensuring that up-stream groups have access to fish by limiting downstream commercial fishing opportunities. Commercial fisheries are never opened on Fridays, for example, to allow more fish into the river system on the weekends when anglers are most active. Current management plans also limit commercial fishing for pink salmon in August and include spatially strategic closures called “conservation corridors” to ensure passage of salmon to rivers and anglers further north.

**Fig. 3:**
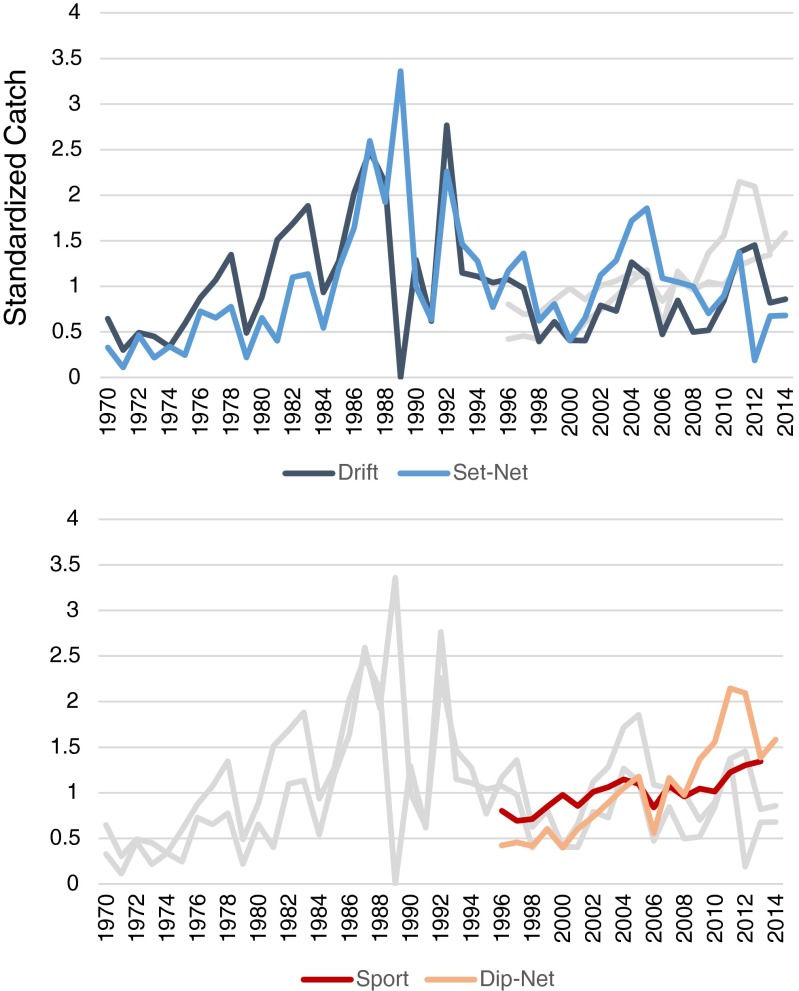
Comparison of catch data for Upper Cook Inlet salmon fisheries. Catch data, standardized, shows relative stability of each fishery over time. It also illustrates year to year variability, and the impacts of key events such as EVOS on the drift fishery in 1989 and the closure of set-net fisheries in 2012

The recent growth of sport and personal use fisheries (Fig. 3) would suggest that the spatial differences (Fig. 2) in combination with strategic closures have, at least historically, been sufficient for limiting the similarity of these fisheries. This is further evidenced by rapid growth of the dip-net fishery following its inception, from roughly 100,000 fish caught in 1996 to over 500,000 in both 2012 and 2013 and without any obvious impact on the catch in other sectors.

Yet, it is also possible that the current abundance of sockeye in the UCI functions as an equalizing mechanism, improving everyone’s access regardless of how, when, or where they fish. Sport interests have long argued for more explicit partitioning of the various fisheries: that sockeye, pink, and chum salmon should be managed for commercial uses while king and coho salmon should be managed for sport fisheries (Mayor’s Blue Ribbon Sportsmen Committee’s 2011). Nevertheless, only now that king salmon runs are seeing notable declines has the conflict reached a point where the sport sector is calling for complete elimination of set-netting in the Inlet.

### Additional Equalizing Mechanisms

State of Alaska law requires equal access to natural resources for all Alaska residents; the state constitution requires that resources be managed to “the maximum use consistent with public interest” (§1), “for the maximum benefit of [all Alaskans]” (§2), and the Alaska Subsistence Law (1978) establishes subsistence uses of fish and game as the highest priority save sustainability. In theory, these pieces of legislation serve as an equalizing mechanism by ensuring that Alaskans will never be displaced from fisheries by corporate interests. In addition, fisheries in the UCI are managed through a two-tiered system: the state’s Board of Fish (BoF) addresses political decisions such as allocation of catches among groups, and the Alaska Department of Fish and Game (ADF&G) addresses scientific issues, such as identification of sustainable yield and escapement targets (ADF&G 2009). The BoF is made up of seven political appointees who make decisions by simple majority. Because of the odd number of members, there is a likelihood that one sector will have more influence than another, meaning that the BoF can serve as an equalizing mechanism among the fishing groups, at least with respect to how each are prioritized through management actions. In recent years, for example, the BoF has had stronger representation for sport fishing interests, which some sport fishers argue is essential as they perceive themselves to be a political underdog by comparison to commercial fisheries (Mayor’s Blue Ribbon Sportsmen Committee’s 2011). Some commercial fishers argue the opposite, however, that a BoF composition that favors sport fishing institutionalizes competitive disadvantage for their sector (Harrison 2013).

### Resilience and Adaptability

As previously noted, resilience and adaptability can provide important equalizing mechanisms in scenarios where different livelihood groups compete for resources. UCI commercial fisheries and drift fishers in particular demonstrated resilience to closures caused by the Exxon Valdez Oil Spill (EVOS) in 1989. Drift fleets were closed for the entire season as a precautionary measure, but east side set-netters were allowed to fish and ended up logging their highest year on record. This created some short-lived animosity between the two commercial fishing groups but the fish processing plants kept operating and the UCI commercial fishery as a whole emerged from the spill relatively unscathed. The drift fleet also recovered immediately from the closure, logging an average catch the very next year and then logging its highest catch on record in 1992 (Fig. 3). Exactly how drift fishers were impacted by the closure is unclear; many drift fishers hold other occupations outside of the fishing season, and this livelihood diversification likely contributed to their resilience.

As already noted, both sport and east side set-net fisheries experienced closures in 2012 because of low king salmon abundance. East side set-net fishers had one day of fishing before being closed for the season, and sport fisheries in the Kenai River were first limited with restrictions on bait, and then closed completely for two weeks to allow kings passage to spawning grounds. Catch data for these fisheries (Fig. 3) show that set-net fisheries were clearly impacted, and they have yet to return to previous levels of productivity, logging below average years in 2013 and 2014. Sport fisheries do not appear to have been similarly affected by the 2012 closure; they logged above average years in both 2012 and 2013 (the most recent year for which there is public data).

**Fig. 4:**
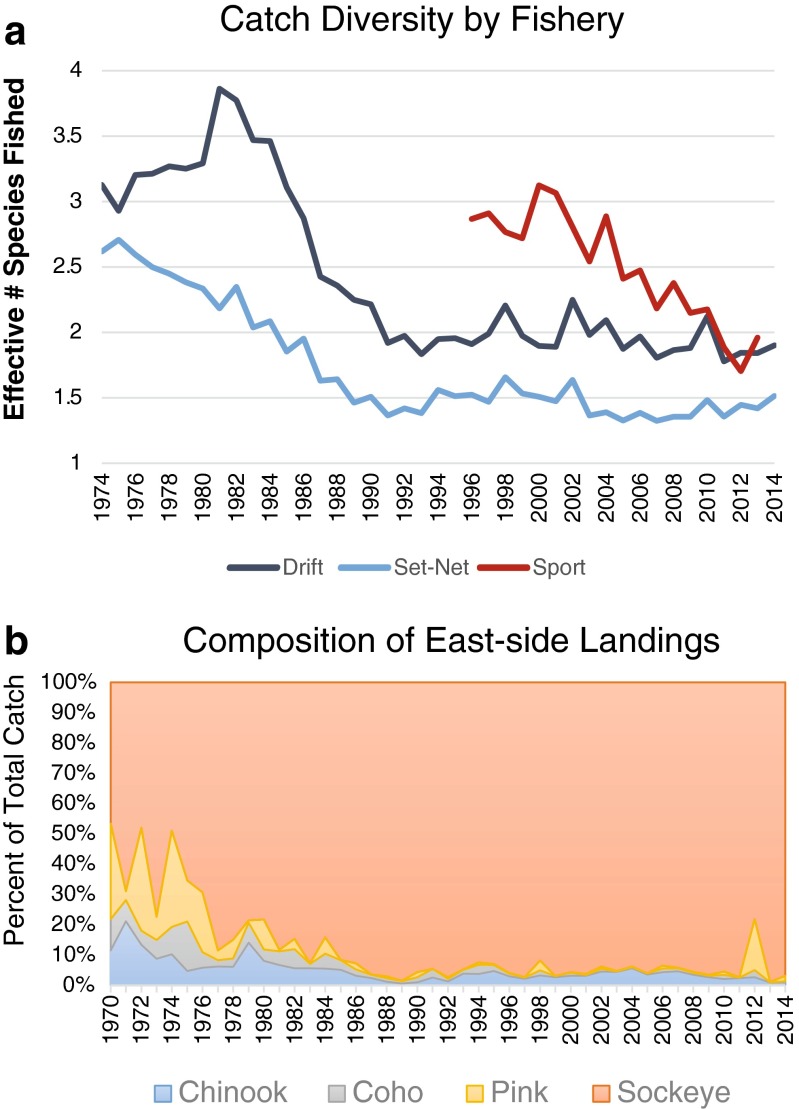
Catch diversity in Upper Cook Inlet fisheries. **a** The effective number of species fished by each fishery, calculated using the Shannon Index (see methods). The set-net fishery shows the greatest decline over time, currently fishing only 1.5 effective species (sockeye salmon, and pink salmon every other year). **b** Catch composition in the east side set-net fishery shows more detail on the decline in catch diversity and the role that pink played for some fishers in 2012

This difference in how the impacts of the 2012 closures were experienced is indicative of differences in resilience and adaptability among fishers of the two fisheries. Catch diversity, for example, is one source of resilience and adaptability for fishers: fishers who focus on fewer species or use highly selective gear tend to be more impacted by and less adaptable to environmental changes (Hamilton *et al.*^2003^; Nesbitt 2014). In the UCI, catch diversity has declined for all sectors over the last 20 years, but the east side set-net fishery is the least diverse of the three (Fig. 4a). The drop in catch diversity for the set-net fishery is a result, in part, of management actions limiting commercial fishing for pink and coho salmon to ensure passage to rivers further north (Shields and Dupuis 2013). Pink salmon are an important secondary catch for east side set-netters, in part because they peak later in the summer than do king and sockeye runs, though their runs are strong only every other year. Some set-netters did fish for pinks late in 2012 to offset the impacts of the closures (Fig. 4b), but these limited opportunities were not enough to keep the set-net fishery from logging a catch 82 % below average that year.

The observed decline in catch diversity in the sport fishery, however, is likely not a sign of reduced resilience because it was driven by an increase in sockeye harvests rather than a decline in the total number of species fished. Sport fishers can target species such as rainbow trout, smelt, and grayling, and managers also have options such as “catch and release” and bait limitations that can be enacted on the sport fishery prior to outright closures.

Finally, because commercial salmon fisheries are managed as limited access, set-net fishers are both restricted to, and financially invested in, highly specialized gear and fixed fishing locations. They cannot easily adapt to changing ecological conditions by fishing elsewhere or modifying their gear or by entering other commercial fisheries, except at great financial costs. Drift fishing permits and the associated equipment currently sell for over $USD 200,000, whereas set-net permits and equipment sell for an order of magnitude less given the fishery’s uncertain future (Alaska Boats and Permits 2015).

### Pluralism and Equity

It is reasonable to infer from the high profile and rancorous nature of conflict in the UCI that pluralism is not the most highly touted value among fishers in the region. However, previous research suggests that the community is not as divided with respect to their values as the ongoing conflict would appear to indicate. A survey of Kenai River dip-netters from around the state shows that a majority believe that there are enough fish to go around and that all of the different fishing groups have a right to fish in the region (Harrison 2013). This research also shows that local residents from all fishing sectors share a core set of priorities about how local salmon fisheries should be managed that include ecosystem and community sustainability and the maintenance of local fishing traditions and culture (Loring *et al.*^2014^). Nevertheless, conflicts in the region have escalated to the point where sport-fishing interests are actively seeking the outright elimination of the east side set-net fishery, and many people are so entrenched in what has become an ideological war over fish that they regularly stereotype and dehumanize one another (Harrison and Loring 2014).

Underlying issues of equity are likely to blame when conflicts escalate to the point where people are willing to take actions that threatens others’ livelihoods (Harrison and Loring 2014). That is, people escalate their own strategies commensurately and to the detriment of others only if they believe that their basic rights are not protected or that the governance system is inherently unfair. In the UCI, both sport anglers and set-netters perceive the system as being stacked against them, with both contending that the other has more political sway and that their right to harvest salmon is being threatened. Sport anglers worry that commercial fishing interferes with their right to fish and also threatens the long-term sustainability of the fisheries, while set-netters contend that they bear an unfair proportion of the costs of management and conservation, and also worry that the sport fisheries, in fact, are threatening the sustainability of king salmon runs through overharvests and undocumented impacts of catch and release fishing. The example of an apparent loss of resilience within the set-net fishery as a result of management actions does support their contention that the impacts of conservation decisions are unequally distributed.

**Fig. 5:**
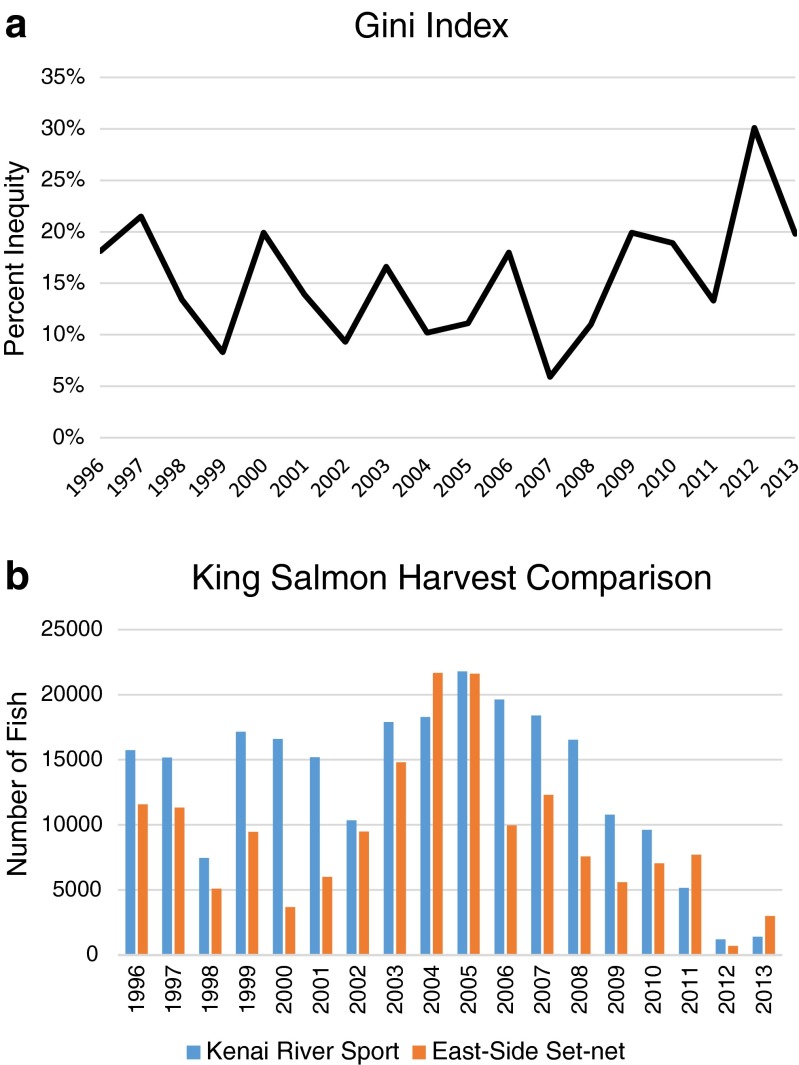
Inequity among fisheries. **a** The increase of inequity, calculated using a GINI index in 2012 is statistically significantly different from the mean. **b** A comparison of the number of king salmon caught by the east side set-net fishery and Kenai River sport anglers

The question of equity in UCI fisheries can also be explored using catch data (Fig. 5). A GINI index for the fisheries, which provides one measure of equity (Voss *et al.*^2014^), shows that while catches have remained relatively equitable over time, inequity doubled in 2012 due to the closures. Similarly, a simple comparison of the number of king salmon caught by east side set-netters and by sport anglers in the Kenai River further suggests that the set-net fishery is indeed bearing an unfair proportion of the conservation costs (Fig. 5b): since 1996, sport anglers in the Kenai River harvested more king salmon than east side set-netters in all but three years: 2004, 2011, and 2013. On average, the ratio of kings caught each year by set-netters to sport anglers is 0.79. In addition, not every king salmon caught in east side set-nets is bound for the Kenai River (Willette *et al.*^2015^). Thus, there is no apparent ecological basis for the east side set-net fishery to be singled out over sport fishing as the primary conservation concern for Kenai River kings, though it continues to bear this reputation in local media (Medred 2013; Caldwell 2015).

## Discussion

The analysis above highlights the various ecological and institutional aspects of UCI fisheries that have fostered the coexistence of sport, commercial, and personal use groups in the region. However, recent declines in king salmon and the escalation of local conflicts make it clear that this coexistence is not stable but bolstered in the past by the sheer abundance of salmon. All three fishing groups make important contributions to the region, economic and otherwise (Knapp 2012; Harrison 2013), so the new political initiative to eliminate set-net fishing would arguably do more harm than good in terms of regional resilience and community well-being. However, as long as locals are mired in this intense conflict and feel that their livelihoods are threatened, it is unlikely that they will come to collectively recognize the value of this biocultural diversity to their communities. That is not to say that conflict need be eliminated outright; conflict can be an important aspect of group maintenance and can be essential for achieving ethical and sustainable outcomes (Spicer 1971; Ominayak and Thomas 2009; Banerjee 2012). Still, conflict can become a pathology that hinders a community’s collective ability to solve emerging problems in an equitable manner (Harrison and Loring 2014).

The question that emerges, then, is what policy actions could enable these various fishing groups to better coexist and cooperate on solving these issues. The analysis above points to multiple options: first, modifications to set-nets may be a way to reduce competition between sport and set-net fisheries, as limited research has shown that king salmon swim deeper than sockeye (Welch *et al.*^2014^; but see Willette *et al.*^2015^). While arguably a more moderate solution than the outright ban on set-nets that some have proposed, this solution would leave unaddressed the question of whether set-net fishers have equal right to fish for king salmon. Likewise, there is presently no scientific basis to suggest that the set-net fishery more than the in-river fishery is responsible for recent declines, so targeting the set-net fishery alone is hard to justify. Thirdly, the change in gear would likely further reduce the resilience of the set-net fishery, so this solution would have to be weighed against any risks that the change would create for set-netters in the future.

A second option is to find ways to improve the set-net fishery’s resilience to closures driven by concerns for king salmon. Specifically, this could be achieved by making management changes that increase the fishery’s catch diversity, for example by allowing more fishing time for pink salmon later in the summer. Pink salmon runs are only fishable every other year, but runs peak at the tail end of king and sockeye season, meaning that strategic fishing in August could improve the set-net catch with minimal impact on king runs (unless the timing of king salmon runs changes to later in the year as well). On average, the price currently paid for pink salmon is just one quarter that for sockeye, but this is an improvement in recent years that makes the pink runs at least commercially viable. Again, this solution would not address the rights-based issues that arguably underlie the conflict, but it would mitigate the impacts of conservation-driven closures on set-net families if and when they are necessary.

It is also arguably beholden on the state to better ensure equity in management and to protect the rights of all involved. As noted above, there is no scientific basis to single out set-nets over in-river anglers, and a case could be made for more long-term in-river restrictions, given that in-river fishing generally catches more kings than the set-net fishery. The proposed set-net ban would instead re-allocate king salmon to sport fisheries based only on the notion that sport fishers should have more right to the fish because they generate more profit. Recently, the Alaska State Superior Court failed to ensure equity for the set-net contingent, arguing that the state is bound only to protect commercial fishing as a single sector. In the judge’s words, “set netters are not a ‘user group’ any more so than sport fishers using fly rods are a distinct user group from those using spinning rods” (Easter 2014: 5). This finding, while congruent with state law (Alaska Statute 16.05.251), is factually inaccurate because as noted above these and most of Alaska’s various commercial fisheries are managed as limited entry, meaning that the set-net fishers displaced by the ban could not easily adapt by adopting new gear and targeting new species. Barring a change to how fisheries are governed, the ban would effectively close set-net families out of commercial salmon fishing in the region entirely.

## Conclusion

If fostering bioculturally diverse communities is a societal goal, and there are strong scientific and ethical arguments for it to be (Maffi 2001; Turner *et al.*^2003^; Kassam 2010; Leslie and McCabe 2013), an essential research question is whether discernable patterns of social, ecological, and economic circumstances can be identified that consistently allow for diverse groups of stakeholders with interests in shared resources to coexist (Ostrom and Cox 2010). In community ecology, CT provides a set of concepts that begin to address these questions. With some additions drawn from the social sciences, I have implemented here a prototype version of CT with social-ecological systems.

Many existing frameworks and theories for understanding conflicts focus on individual behavior and the material and economic aspects of the conflict, for example game theory or the typology of conflict suggested by Charles (1992; see also McClanahan *et al.*^2013^). Yet, conflicts are often complex emergent phenomena that defy such typologies and often transcend the specifics observed in individual disputes (Harrison and Loring 2014). As I illustrate above, SCT as proposed here draws focus to functional relationships at the system level, and the UCI case study shows that SCT is informative, at very least, as a heuristic for diagnosing the ecological and institutional dimensions of conflict in a shared landscape or seascape.

The discussion above also illustrates how SCT can inform a policy framework for pursuing sustainable and just outcomes in a contested setting. SCT holds that groups will only achieve stable coexistence if the similarity of their niches are sufficiently differentiated, and, perhaps more importantly, if competition among groups is limited such that no one group is capable of or willing to eliminate another. Resilience, adaptability, equity, and pluralism are all outcomes that can be actively fostered either by individuals or governing institutions, but a persistent challenge with putting concepts like resilience into practice is that they require an explicit normative plan (Lélé and Norgaard 1996; Brand and Jax 2007). Many undesirable systems are highly resilient, and in social systems, resilience can often come at significant social cost (Oliver-Smith 2013). Coexistence and biocultural diversity provide a compelling normative strategy for building social resilience, providing an answer, in other words, to the persistent question, “resilience of what” (Carpenter *et al.*^2001^)?

As discussed earlier, one must be cautious when applying natural science concepts to social systems. Even where analogies are warranted, their limits must be understood. SCT as set out here offers concepts for mapping the ecological and institutional dimensions of intergroup dynamics, specifically as they relate to scenarios of conflict over shared resources. It does not purport to explain the behavior of actors in these scenarios or how these social-ecological systems came to be in the situations that they are in. Finally, it does not attend to mutualistic rather than competitive scenarios, though this would be a fruitful area for future research and development of the theory.

These caveats notwithstanding, the case study above shows that SCT holds promise for helping us learn about what conditions are necessary for effective coexistence among otherwise competing groups of people. Future research could explore how SCT might be used in tandem with existing frameworks for the analysis of common pool resource systems (e.g., Ostrom 2009; Cox 2011). These frameworks, such as Ostrom’s (2007) Social Ecological Systems (SES) framework, have proved powerful for amassing detailed empirical data on the social and ecological features of resource systems from around the world (Cox 2014). Understanding how the components of these systems interact is a theoretical question, and one that Ostrom and Cox (2010) argue is a next step for commons research. SCT could inform compelling hypotheses for why patterns such as sustainability and coexistence or non-compliance and overharvest emerge from the bricolage of social institutions, ecological circumstances, and social and ecological histories that sustainability researchers invariably encounter.

## Data Analysis Methods

Standardized catch for all UCI fisheries is calculated by diving the annual total catch (in number of fish) by the average of totals for all years on record.

Catch diversity is calculated as the effective number of species fished using a Shannon index:

$$ {e}^{{\displaystyle {\sum}_{i=1-n}^S{p}^i\times \ln {p}^i}} $$

where *S* is the number of species caught and *p* is the ratio of catch per species to the total catch in a given year. For commercial fisheries, a 5 year average was used in order to account for the two-year periodicity of pink salmon returns. This mutes the signal of inter-annual variability in favor of illustrating longer trends.

Change in catch for the set-net fishery is shown as proportion of value contributed by species, corrected to 2014 USD. Landings value is calculated using the average prices paid and average species weights listed in Shields and Dupuis (2014, their Appendix B11 and B12).

The GINI index is calculated for each year since 1996 with the following formula,

$$ G=\frac{n+1}{n}-\frac{2{\displaystyle {\sum}_1^n\left(n+1-i\right){x}_i}}{n{\displaystyle {\sum}_1^n{x}_i}} $$

where *x* is standardized catch for each fishery in a given year ordered from least to greatest.
